# New Methods of Reconstruction for Old Challenges: The Use of the Integra Graft in Necrotizing Soft Tissue Infections of the Male Genitalia

**DOI:** 10.1155/2021/5777235

**Published:** 2021-10-07

**Authors:** Katharina Mitchell, Chad Crigger, Chad Morley, John Barnard, Vidas Dumasius

**Affiliations:** ^1^West Virginia University Department of Urology, USA; ^2^Marietta Memorial Hospital Department of Cosmetic, Plastic and Reconstructive Surgery, USA

## Abstract

In this paper, we describe two cases of Fournier's gangrene (FG) in which Integra grafting was used for reconstruction. FG is a progressive necrotizing infection occurring in the perineal region and on the external genitalia. Reconstructive options using local tissue are limited due to the destruction this infection imposes on the soft tissue. Integra graft is a bilaminate artificial dermis made of shark chondroitin 6-sulfate and bovine collagen. It is applied to the wound bed after debridement and establishment of a healthy, well-vascularized wound base. The patients in this case series had large defects which could not be closed primarily with tissue beds and would not have been appropriate for skin grafting. Therefore, an Integra graft was placed. In both patients, the wound beds were appropriate for skin grafting after three weeks. Without the Integra graft, both of our patients would have needed to wait a considerable amount of time prior to reconstruction. Our case series further illustrates and supports the use of Integra grafts in such a scenario following Fournier's gangrene which has only previously been published on three occasions, all of which demonstrated successful outcomes.

## 1. Introduction

Fournier's gangrene (FG), often also referred to as necrotizing fasciitis, is a progressive necrotizing infection occurring in the perineal region and on the external genitalia [1]. The underlying pathology of FG is an obliterative endarteritis of the subcutaneous arteries which leads to ischemia, destruction, and subsequent gangrene of the overlying skin and subcutaneous tissues. It is a rare, but highly lethal, process due to severe multiple organ failure. The incidence is more common in men, persons over 50 years, those with diabetes mellitus, long-term alcoholics, and people with malignant neoplasms [1, 2].

Often the nidus for this synergistic polymicrobial infection is found in the genitourinary tract, lower gastrointestinal tract, and/or skin [1]. Though often polymicrobial, *Proteus mirabilis* has been found to be the predominant pathogen [3]. Standard of care in treating this disease process begins with rapid and aggressive surgical debridement of necrotic tissue, hemodynamic support, and broad-spectrum parental antibiotics. Following radical debridement, sterile dressings and negative pressure wound therapy (wound vacuum devices) are typically used to treat open wounds [1].

Reconstructive options using local tissue are limited due to the destruction this infection imposes on the soft tissue, which is often too inflamed or lacking altogether due to the extensive surgical debridement required. These factors limit reconstructive options based on local tissue transfers or utilization of adjacent fasciocutaneous flaps. Tissue flaps in turn can also be problematic—either too bulky or insufficient coverage if skin grafting alone is utilized [3]. A new use for existing technologies may fill the void. The Integra graft (Integra LifeSciences, Plainsboro, NJ) is a bilaminate artificial dermis made of shark chondroitin 6-sulfate and bovine collagen. It is applied to the wound bed after debridement and establishment of a healthy, well-vascularized wound base. Vascularization occurs in approximately two or three weeks after application, which creates a neodermis which can be skin grafted [3]. An Integra bilayer has been used in many other reconstructive modalities since it first was approved in 2004. However, there are very few cases which report using Integra for scrotal and/or penile reconstruction following Fournier's gangrene. We present two cases which highlight the use of the Integra graft to initiate scrotal and penile reconstruction following extensive debridement of Fournier's gangrene.

## 2. Case Reports

### 2.1. Case 1

**Figure 1:**
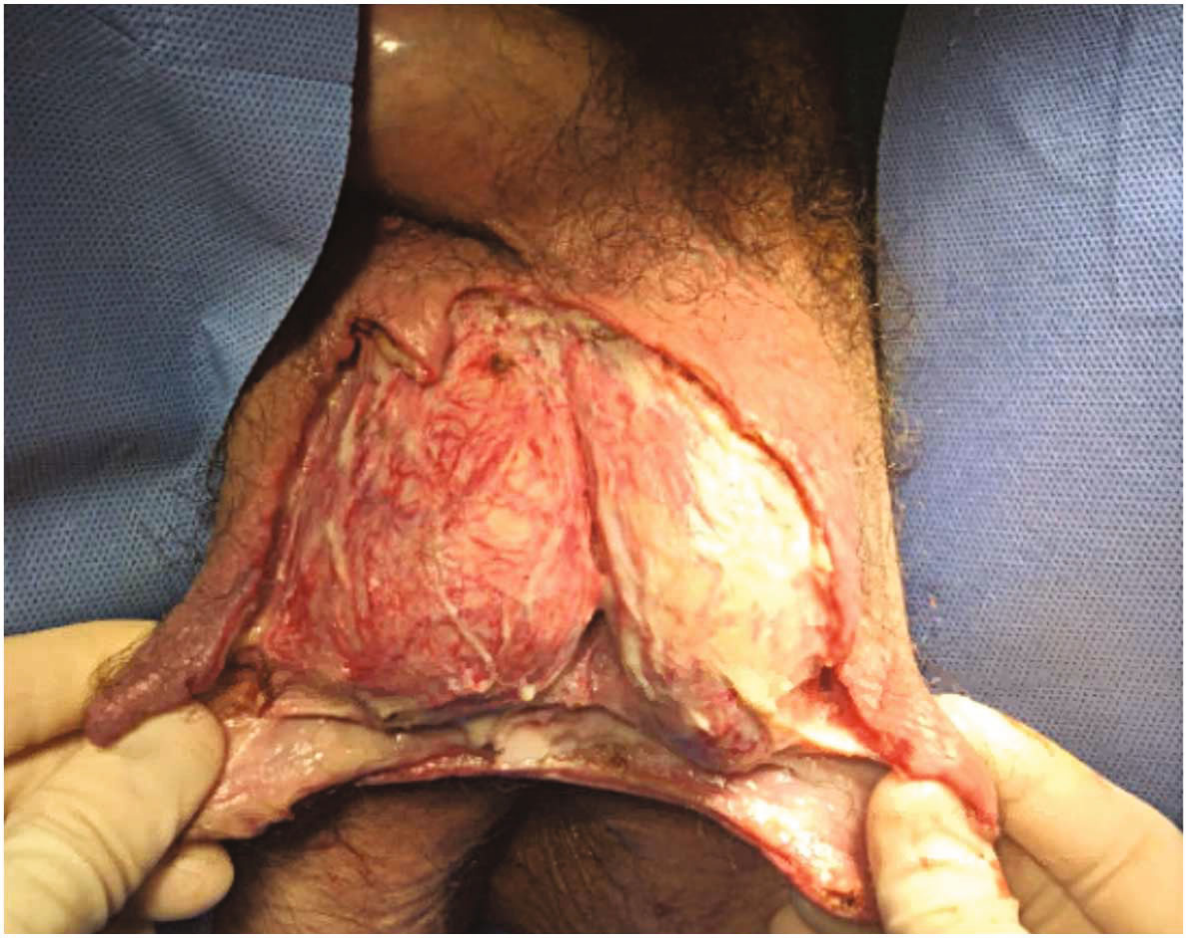
Scrotal wall wound measurement 12 cm (width) × 11 cm (length). Right base of penis wound measuring 3 cm (width) × 4 cm (length) × 3 cm (depth). Intraoperative during debridement.

**Figure 2:**
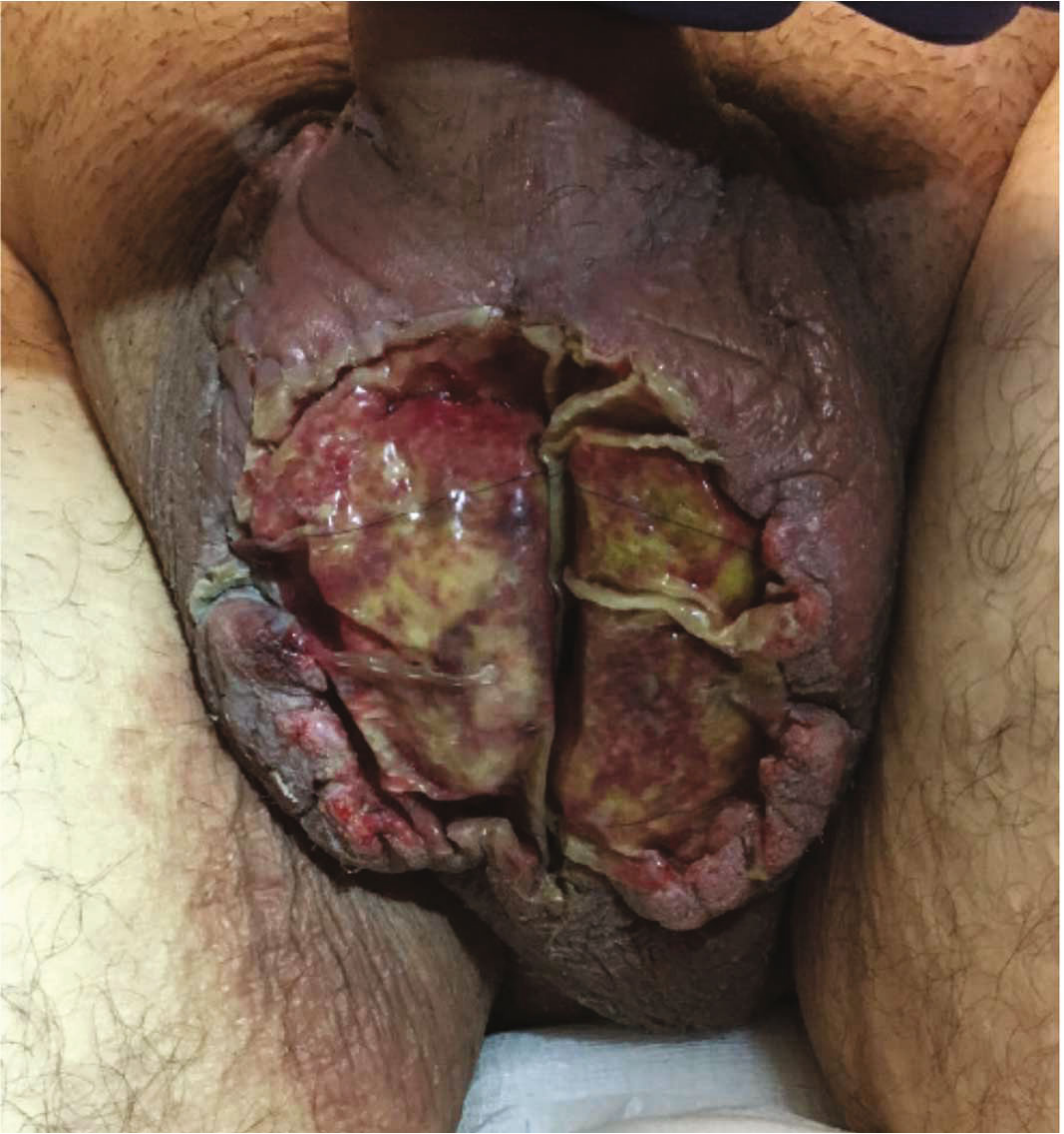
POD 3 after wound vac was removed. Integra graft seen to have taken well.

A 43-year-old Caucasian male with a past medical history of poorly controlled diabetes mellitus type 2 confirmed by most recent hemoglobin A1C of 13%, history of obstructive sleep apnea (OSA), and remission of polycythemia vera presented with scrotal pain, edema, and rash, after noticing what he believed was a yeast infection of his skin. A necrotic eschar was noted on his scrotum. Computed tomography (CT) revealed gas gangrene concerning necrotizing soft tissue infection. He was started on vancomycin, Zosyn, and clindamycin and then underwent emergent scrotal and penile skin subcutaneous tissue incision and drainage. Subcutaneous tissue was noted to be edematous and skin without blood flow. The necrotic skin was sharply excised until the edges bled; this resulted in a large portion of the scrotal skin being removed. He was admitted to the surgical intensive care unit (SICU) in stable condition. On hospital day three, he returned to the operative suite for a second exploration of the wounds with washout and further debridement (Figure 1). Patient treatment continued with broad antimicrobial coverage along with local wound care. After coordinating with our plastic surgery colleagues, he was taken back to the operating room on hospital day eight for further sharp debridement of open scrotal and perineal wounds, complex perineal wound closure, and placement of an Integra graft covering the scrotal wound measuring 11 × 9 cm (Figure 2). An additional Integra graft measuring 10 × 12.5 cm was used to cover the perineal wound followed by placement of wound vac in place for 72 hours.

Twenty-one days after his last surgery with urology, he returned to the operating theater with plastic surgery. At this time, he underwent a 13 × 14 cm full-thickness skin graft which was obtained from the patient's suprapubic region which was then thinned out, and fibrous tissue was removed. Next, the scrotal area was debrided with a curette and scraping pad; once bleeding was observed, both the scrotal area tissue and harvested tissue were copiously irrigated with sterile saline; the skin graft was sutured to the scrotal defect with openings made for drainage of the serous fluid. Finally, a wound vacuum device was placed over top. He was discharged home on a two-week course of oral antibiotics, and at his 1-month follow-up, his scrotal wound with a skin graft was well healed.

### 2.2. Case 2

**Figure 3:**
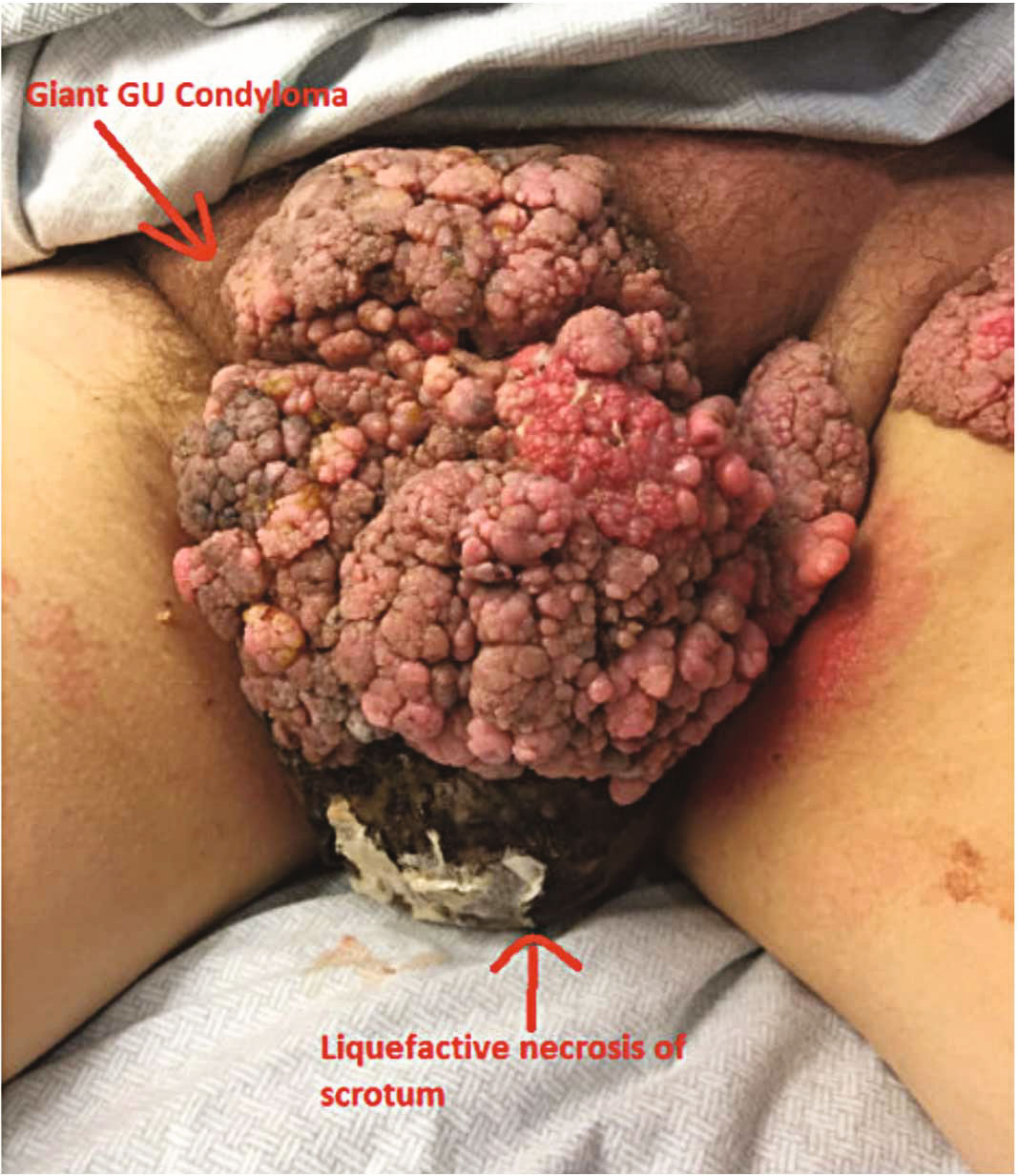
A patient's presentation with giant GU condyloma and liquefactive necrosis of the scrotum.

A 59-year-old male with a past medical history of chronic obstructive pulmonary disease (COPD), hyperlipidemia, diabetes mellitus type 2, and coronary artery disease (CAD) requiring multiple coronary stents and on Plavix presented with a giant condyloma involving his penis and perineum for greater than twelve years (see Figure 2). He arrived as a transfer from the outside facility with Fournier's gangrene. On presentation, his Fournier's gangrene severity index (FGSI) was 10. Preoperatively, he carried several diagnoses including Fournier's gangrene, sepsis, giant genital condyloma, and left inguinal hernia (Figure 3). Prior to surgical debridement, he was placed on broad-spectrum antibiotics.

Intraoperatively, he underwent incision and drainage of the penis and scrotum, complete scrotectomy, right orchiectomy, left orchiectomy, and penile circumcision. With exploration, the patient had extensive genital condyloma covering his suprapubic region, penis, scrotum, perineum, and perianal area with Fournier's gangrene of the scrotum extending proximally and involving the suprapubic region. All necrotic tissue was excised down to the abdominal fascia which was inspected and uninvolved. Bilateral testicles were necrotic, and bilateral orchiectomy was performed as a result. General surgery was consulted and evaluated the perineum, rectum, and left inguinal hernia. Postoperatively, he was taken to the SICU in stable condition for medical optimization and management of his complex wounds.

**Figure 4:**
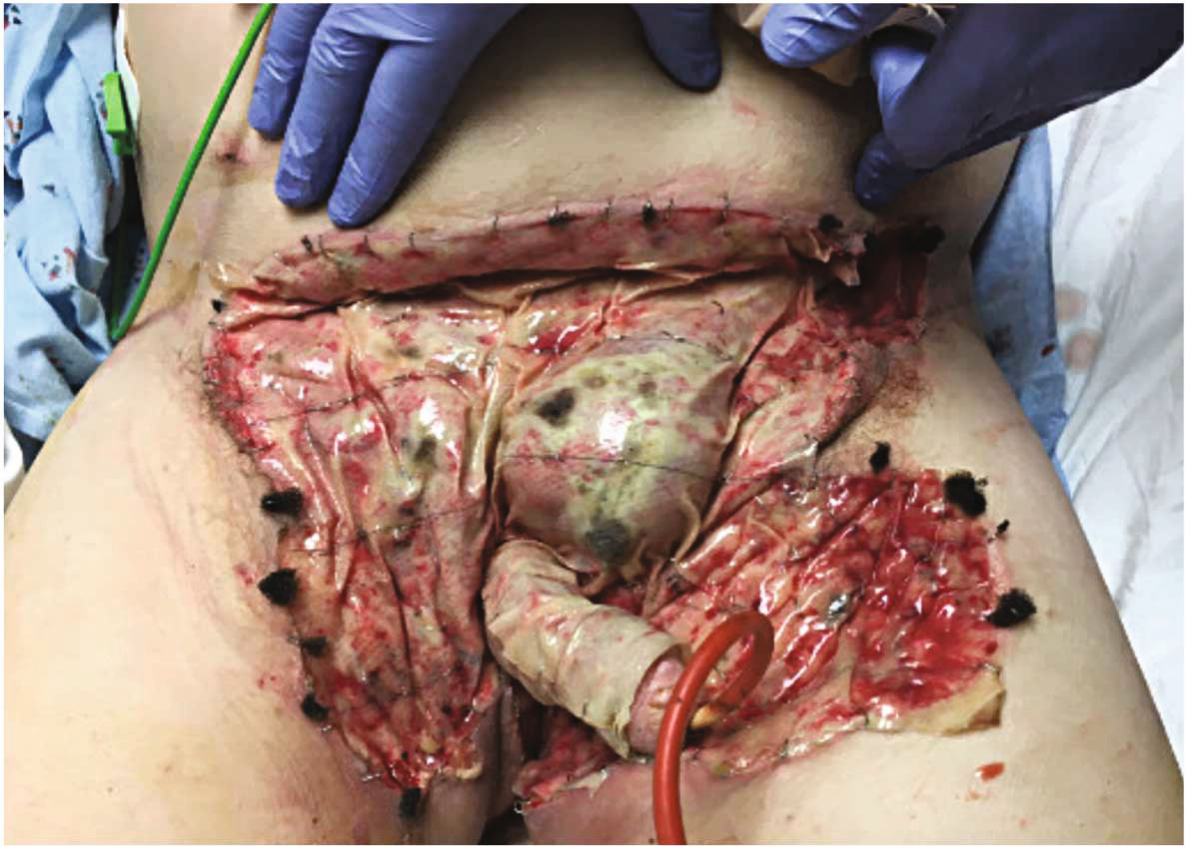
POD 7 from incision and debridement of the penis and scrotum, scrotectomy, and bilateral orchiectomy. POD 2 from colostomy creation, excision of condyloma, sharp debridement with Integra graft application, and wound vac placement. Photo taken after wound vac was removed.

Six days after his initial surgery, he returned to the operating room with general surgery and plastic surgery. General surgery performed laparoscopic inguinal hernia repair for his left inguinal hernia, laparoscopic transverse loop colostomy, and open excision of bilateral thigh and groin condyloma. Simultaneously, plastic surgery performed sharp debridement of the lower abdominal wall and genital area with application of an Integra graft in addition to a traditional wound vacuum device (Figure 4). Fourteen days after surgery, he was discharged home. At his postoperative visit at 5 weeks, the Integra graft was noted to be well incorporated to the lower abdomen and penis.

Final pathology from the patient's bilateral thigh condylomas and scrotal sac was significant for condyloma acuminata with focus of high-grade intraepithelial carcinoma in the scrotal sac (with negative margins). Despite review of pathology and further reconstructive options offered to the patient at that point, such as full-thickness skin graft, the patient declined any further surgical intervention. The patient passed away shortly after.

## 3. Discussion

Fournier's gangrene is an ominous diagnosis associated with a high mortality and even greater morbidity that is often prolonged. In the cases provided, our patients shared similar characteristics—obese males with poorly controlled type 2 diabetes mellitus. While these characteristics are often found in the “index patient” with Fournier's gangrene, the disease has an incidence of just 1.6 cases per 100,000 men per year. Demographically, the mean age is 50.9 years with the disease substantially more common in men with a male-to-female ratio of 10 : 1 [2]. Additionally, diabetes mellitus is present in 20–70% of the patients who present with Fournier's gangrene [3]. It is worth mentioning that both our cases were diagnosed clinically, which is the mainstay of diagnosis, with imaging being reserved during instances of clinical uncertainty [3]. While reconstructive options are often limited due to lack of available healthy tissue, both our cases demonstrate stable wound coverage using Integra grafting. Furthermore, coverage with the Integra bilayer minimizes patient discomfort due to repeat dressing changes, while avoiding immediate reconstruction with possibly compromised patient tissue and recovering from major surgical insult. Additionally, the Integra bilayer allows for the necessary time to optimize patient nutritional-metabolic status prior to final reconstruction.

While our case series focuses on the use of Integra grafts in the reconstruction of Fournier's gangrene, many other methods have been more frequently used for reconstruction. A study analyzing aggregate data from 16 studies regarding Fournier's gangrene reconstruction found the following methods to be implemented with these corresponding frequencies: secondary intention with delayed primary closure 10.4%, implantation of the testicle in a medial thigh pocket 8.5%, loose wound approximation 1.4%, skin grafts 22.6%, scrotal advancement flaps 16.0%, flaps 30.1%, and flaps or grafts in combination with tissue adhesives 5.2% [4]. Each method of reconstruction has different implications as well as demands on the healthcare system and patient. For example, healing by secondary intention is used in patients with a small postsurgical defect confined to the scrotum. In the case of Fournier's gangrene, inflammation and infection of the surrounding tissue limit the reconstruction techniques possibly—our patients had large defects which could not be closed primarily with tissue beds which would not have been appropriate for skin grafting. Therefore, an Integra graft was placed. In both patients, the wound beds were appropriate for skin grafting after three weeks. Without the Integra graft, both of our patients would have needed to wait a considerable amount of time prior to reconstruction.

Our case series further illustrates and supports the use of Integra grafts in such a scenario following Fournier's gangrene which has only previously been published on three occasions, all of which demonstrated successful outcomes [3, 5, 6]. Successful outcomes were determined based on a follow-up at 1-3 months postop with patients demonstrating complete wound healing with good functional and aesthetic results. Indeed, larger aggregate studies following patients who have undergone scrotal reconstruction using Integra grafts in the setting of Fournier's gangrene are needed to better understand the long-term outcomes associated with the use of Integra grafting for genitourinary reconstruction.
